# Are acceptance rates of a national preventive home visit programme for older people socially imbalanced?: a cross sectional study in Denmark

**DOI:** 10.1186/1471-2458-12-396

**Published:** 2012-06-01

**Authors:** Yukari Yamada, Anette Ekmann, Charlotte Juul Nilsson, Mikkel Vass, Kirsten Avlund

**Affiliations:** 1 Section of Social Medicine, Department of Public Health, University of Copenhagen, Oster Farimagsgade 5, DK-1014, Copenhagen, Denmark; 2 Department of Social Medicine and Health Policy, Faculty of Medicine and Dentistry, Palacky University Olomouc, Hnevotinska 3, 77515, Olomouc, Czech Republic; 3 Danish Aging Research Center (DARC), University of Aarhus, Odense and Copenhagen, J.B. Winslows Vej 9B st. tv, 5000, Odense, Denmark; 4 Centre for Healthy Aging (CEHA), University of Copenhagen, Oster Farimagsgade 5, DK-1014, Copenhagen, Denmark; 5 Research Unit and Section of General Practice, Department of Public Health, University of Copenhagen, Oster Farimagsgade 5, DK-1014, Copenhagen, Denmark; 6 Section of Health Services Research, Department of Public Health, University of Copenhagen, Oester Farimagsgade 5, DK-1014, Copenhagen, Denmark

**Keywords:** Community dwelling older people, Preventive home visits, Socioeconomic status, Social inequality, Invitational procedure

## Abstract

**Background:**

Preventive home visits are offered to community dwelling older people in Denmark aimed at maintaining their functional ability for as long as possible, but only two thirds of older people accept the offer from the municipalities. The purpose of this study is to investigate 1) whether socioeconomic status was associated with acceptance of preventive home visits among older people and 2) whether municipality invitational procedures for the preventive home visits modified the association.

**Methods:**

The study population included 1,023 community dwelling 80-year-old individuals from the Danish intervention study on preventive home visits. Information on preventive home visit acceptance rates was obtained from questionnaires. Socioeconomic status was measured by financial assets obtained from national registry data, and invitational procedures were identified through the municipalities. Logistic regression analyses were used, adjusted by gender.

**Results:**

Older persons with high financial assets accepted preventive home visits more frequently than persons with low assets (adjusted OR = 1.5 (CI95%: 1.1-2.0)). However, the association was attenuated when adjusted by the invitational procedures. The odds ratio for accepting preventive home visits was larger among persons with low financial assets invited by a letter with a proposed date than among persons with high financial assets invited by other procedures, though these estimates had wide confidence intervals.

**Conclusion:**

High socioeconomic status was associated with a higher acceptance rate of preventive home visits, but the association was attenuated by invitational procedures. The results indicate that the social inequality in acceptance of publicly offered preventive services might decrease if municipalities adopt more proactive invitational procedures.

## Background

Preventive home visits in Denmark have been offered to all citizens aged 80 years or older since 1996 and to all citizens aged 75 years or older since 1998 [1]. The aims of the visits are to give older people feelings of security and well-being, to give advice and guidance about activities and possibilities for support and to facilitate that the older people make better use of own resources and sustain their functional ability for as long as possible. However, it is up to the older people to accept or decline this municipality offer and it is known that only about 60% actually receive preventive home visits. Knowledge about factors that influence acceptance of preventive home visits is, therefore, important to maximize the possible effects of the visits.

In a Danish intervention study on preventive home visits conducted in 1998-2002, several psychological characteristics such as reporting low life satisfaction were found to be related to acceptance of preventive home visits among men [2]. However, the role of socioeconomic status (SES) for the acceptance is not known, although studies have shown that those in low SES are much less likely to accept or participate in other publicly offered preventive interventions [3-8].

Interestingly, it has also been shown that varying invitational procedures to preventive home visits chosen by each municipality had influence on older person’s decision to accept or decline the preventive home visits [9]. Older people who received an invitational letter with a proposed date for the visits accepted the visits more often than those who received other forms of invitations. A possible social inequality in acceptance of preventive home visits may therefore be alleviated by the invitational procedures, which are remediable.

The aims of this study were to analyse whether there was an association between SES and acceptance of preventive home visits in a sample of community-dwelling older people, and whether invitational procedures to the preventive home visits modified the possible association.

## Methods

### Study population

This study was based on secondary analysis of data from the Danish intervention study on preventive home visits, which had the main aim to examine whether education of home visitors and GPs was associated with functional ability of older people. The study was randomised at municipality level and outcome was measured at individual level [10,11]. Altogether, 5,788 non-institutionalised older people living in 34 Danish municipalities born in 1918 (80-year-old at baseline) or 1923/24 (75-year-old at baseline) were invited to participate in the baseline study conducted in 1998-1999. Written consent was obtained from 4,060 persons (participation rate 70.1%). The study showed that the intervention was associated with beneficial effects on functional ability, especially among 80-year-olds [11] and among women [12,13].

The present study was restricted to the 80-year-olds (N = 1,184), because many of the 75-year-olds had not yet been offered a preventive home visit when the baseline study was conducted (1998/1999). Mailed questionnaires at baseline were linked to registers at the Statistics Denmark by participants’ civil registration numbers. Analyses in this study were based on participants with full records on all relevant variables (N = 1,023). Thus, the participation rate in this particular study was 57.3% among those who were initially invited to participate in the original intervention study.

### Variables

Acceptance of preventive home visit was measured by a question included in the mailed questionnaire at baseline: ‘Have you accepted the invitation from the municipality to receive a preventive home visit?’ (yes/no).

SES was measured by information on financial assets in 1999 on bonds, stocks, and mortgage deposited in financial institutions, funds on deposit in financial institutions, and debt for financial institutions and mortgage credit debt. They were obtained from registry data from the Statistics Denmark. The financial assets variable was dichotomized distinguishing the 20% with the lowest (-1,095,514USD to 2,731USD) and the 80% with the highest (2,731USD < to 3,649,264USD). This dichotomized variable was used because 1) preliminary analyses with a trichotomous variable showed that patterns of associations were nearly the same for persons with intermediate and high financial assets, 2) the largest contrast between financial assets-categories when predicting poor health has been seen between the lowest end of the assets distribution and the remainder [14], and 3) this dichotomization has been useful in other analyses using the same data finding a clear social inequality in mobility onset among older people [15] .

Three different invitational procedures were identified based on careful investigation on copies of the invitational letters used in 23 municipalities and telephone surveys conducted in the remaining 11 municipalities [9]: *‘Letter with date’,* means that the municipality sent a letter to the older people proposing a date and time for the visit and the older people had to actively contact the municipality if they wanted to decline the visit,*’ Letter without date’* implies that, after receiving a letter from municipalities, the older people had to make a phone call to the municipality for making an appointment, and ‘*Telephone call’* means that no letter was sent to the older people, but that the visit was proposed by a telephone call. One municipality could not provide this information due to change of the employee in charge; consequently older people in this municipality were excluded from the analyses. Since ‘letter with date’ was significantly associated with accepting preventive home visits compared to the other two procedures [9], ‘letter without date’ and ‘telephone call’ were combined into one category in this study.

Gender was included in the analyses as a potential confounder. Mobility disability and psychosocial characteristics were related with acceptance of preventive home visits [2,9]. These variables, however, were not included as covariates, as they were potentially on the pathway between SES and acceptance of preventive home visits. Thus, they can be regarded as possible mediators and should not be adjusted for. In addition, cohabitating status was not included, as earlier analyses showed that it was not related to acceptance of preventive home visits [2].

### Statistical analyses

First, we analysed for an association between financial assets and acceptance of preventive home visits. Second, we analysed how invitational procedures influenced on the acceptance rates. Third, to study a possible modifying effect of invitational procedure on the association between financial assets and acceptance of preventive home visits [16], we made a combined variable of financial assets and invitational procedure with four categories: (1) low assets and ‘telephone or letter without date’ (reference category), (2) low assets status and ‘letter with date”, (3) high assets and ‘telephone or letter without date’, and (4) high assets and ‘letter with date’. All analyses were investigated by logistic regression analyses, adjusted for gender.

Logistic regression analyses were performed using the IBM SPSS statistics version 19. Model 1 describes the crude association between each of financial assets, invitational procedures, and the combined variable of financial assets and invitational procedures with acceptance of preventive home visits. Model 2 was adjusted by gender.

## Results

Forty-nine per cent of the study population accepted the municipality offer of preventive home visits. Combining financial assets and invitational procedures showed that about a half having high financial assets and being invited by a ‘letter with date’ (Table 1).

**Table 1:** Distributions of gender, acceptance of preventive home visits, financial assets and invitational procedures in the sample population (n = 1,023)

Characteristics	N(%)
**Gender**	
Male	428(42)
Female	595(58)
**Acceptance of preventive home visits**	
Accept	498(49)
Decline	525(51)
**Level of financial assets**	
Low	205(20)
High	818(80)
**Invitational procedures**	
Telephone or letter without date	378(37)
Letter with date	645(63)
**Financial assets and invitational procedures**	
Low assets and ‘telephone or letter without date’	72(7)
Low assets and ‘letter with date’	133(13)
High assets and ‘telephone or letter without date’	306(30)
High assets and ‘letter with date’	512(50)

Those with high financial assets had significantly higher odds ratios (adjusted OR = 1.47 (95%Cl = 1.08-2.00)) of accepting the preventive home visit compared to persons with low financial assets. Further, those who had been invited by ‘letter with a date’ were more likely to accept the preventive home visits compared to those who had been invited by ‘telephone or letter without date’ (adjusted OR = 1.73 (95%Cl = 1.34-2.24)) (Table 2).

**Table 2:** Odds ratios (95% confidence interval (CI)) for acceptance of preventive home visits by financial assets and invitational procedures (n = 1,023)

Cases with		Model1	Model2
	outcome/n	(95%Cl)	(95%Cl)
**Level of financial Assets**			
Low	85/205	1.00	1.00
High	413/818	1.44 (1.06-1.96)	1.47 (1.08-2.00)
**Invitational Procedures**			
Telephone or letter without date	152/378	1.00	1.00
Letter with date	346/645	1.72 (1.33-2.23)	1.73 (1.34-2.24)

Model 1: Crude Model.

Model 2: Adjusted for gender.

Analyses with the combined variable revealed that the low assets-group who received the invitation to the preventive home visit by ‘letter with date’ had a marginally significantly larger chance of accepting the preventive home visit compared with persons who received the invitation by other procedures. The same pattern was seen among the high assets-group (Table 3).

**Table 3:** Odds ratios (95% confidence interval (CI)) for acceptance of preventive home visits by combined variable of financial assets and invitational procedures (n = 1,023)

Cases with		Model1	Model2
	outcome/n	(95%Cl)	(95%Cl)
Low and ‘telephone or letter without date’	25/72	1.00	1.00
Low and ‘letter with date’	60/133	1.55(0.85-2,80)	1.54(0.85-2,78)
High and ‘telephone or letter without date’	127/306	1.33(0.78-2.28)	1.35(0.79-2.31)
High and ‘letter with date’	288/512	2.38(1.42-3.98)	2.43(1.45-4.07)

Model 1: Crude Model.

Model 2: Adjusted for gender.

It is of interest that the odds ratio for accepting the preventive home visit was larger among the low assets-group invited by ‘’letter with date’ than among the high assets-group invited by other procedures, though these estimates had wide confidence intervals.

## Discussion

This study analysed for socioeconomic differences in acceptance of preventive home visits and for modifying effects of municipality invitational procedure for the preventive home visits. In summary, this study showed that high SES was associated with higher acceptance rate of preventive home visits. The findings further suggested that the more proactive invitational procedure chosen by municipalities might reduce the negative effect of low SES on the acceptance rate.

A major finding was that persons in low SES were less likely to accept the preventive home visits compared with persons in high SES. This is in line with a recent British review, which concluded that in public tax-financed health systems like Denmark there is a general tendency that preventive services are more often used by people in high SES [17]. Likewise studies on cancer screenings have shown a SES disparity in participating in such preventive strategies in countries with universal insurance coverage [3-8]. It has also been shown that a larger proportion of receivers of the lowest social benefits have refrained from buying medicine and visiting dentists than people who had resources from other sources [18].

The findings of social inequality in accepting preventive home visits may have several explanations. Maybe the older person’s earlier experiences with the social and health care system had an influence: e.g. earlier contact with the municipalities, expectations on possibilities for help and knowledge about what the municipality can offer [19]. Here the user's education, social status and communication skills may play a critical role. It is also possible that the preventive home visits are organized in a way, which is more focused towards the middle class. This supports findings from other health promotion studies, where preventive education was more easily understood by the middle class than by more socially disadvantaged groups [20,21]. Maybe the organization of the preventive home visits should be more targeted to different groups in order to be attractive to all social groups. A first step could be to educate the preventive home visitors about social inequality in health and functional ability. A second step could be to interview older people in different social groups about how to make the preventive home visits the best way. This might give new ideas on how to be better at aiming the preventive home visits to older people in different social groups [20,22].

A second major finding was that the association between SES and acceptance of preventive home visits was attenuated by invitational procedures to the preventive home visits. This means that a larger proportion of older people in low SES would accept the preventive home visits, if the invitation were a letter with a proposed date. This is in line with the careful attention on invitational procedures as an important strategy to increase participation rates among disadvantaged populations in community health promotion and community-based research [22-26].

Even though this study was set up in a specific setting with a government-funded home visit program, our findings were, thus, much in line with studies on other preventive interventions. Since increasing participation rates has been shown to be essential in any preventive interventions, we do think that our findings that more proactive invitational procedure might decrease a social inequality can be translated to other preventive programs, and thus be useful in more general terms with regard to prevention.

We did consider a range of potential confounders of the association between SES and acceptance of preventive home visits, especially mobility disability, which is a good measure of health status of this age group, and psychosocial factors. These variables might be situated in the causal sequence between SES and acceptance of preventive home visits and might therefore be mediators. Adjusting for mediators could lead to an underestimation of the studied effect. Therefore, we chose not to include these factors in our final analyses [27]. We are aware that these factors might explain some of the combined effect of SES and invitational procedure on acceptance of preventive home visits. Yet, it was not an aim of this study to identify explanatory mechanisms.

One limitation of our study was the non-validated question of the outcome measure: “Have you accepted the invitation from the municipality to receive a preventive home visit?”. There was no means to test reliability of this answer; therefore it is possible that some older people may have misunderstood the invitation as an invitation to other forms of home visits such as home help services, thus giving a risk for misclassification.

A possible impact on study results by participants excluded due to missing values (n = 161) is also a limitation. They were almost identical with the included 1,023 regarding distributions of financial assets, and acceptance of preventive home visits, but were significantly different as regards invitational procedures. Only 47% of the excluded received ‘letter with date’, compared to 63% of the included (chi square p = 0.034). This was because more people of those excluded due to missing values on acceptance of home visits had ‘telephone call or letter without date’ as the invitational procedures. It is reasonable to assume that older people who received those invitational procedures, where they did not need to do further action to decline, had a greater probability of forgetting whether they had the offer. If they had declined more often than the included people, which is more likely, the odds ratios of invitational procedures on acceptance of preventive home visits might be larger than estimated in this study.

Strengths of this study were both the robustness of the study population and the possibility to use comprehensive registry data of financial assets as a measure of SES. Self-reported income is most often used in studies investigating social differences in health and health behaviours [28-30], but it is also pointed out that self-reported individual-level SES had poor agreement with aggregate-level from census data [31]. In contrast, under the civil registration system in Denmark, all aspects of private finances connected with financial organizations are captured and furnish vital statistic to be available for research purposes.

## Conclusion

High socioeconomic status was associated with a higher acceptance rate of preventive home visits, but the association was attenuated by municipality invitational procedures to preventive home visits. The results indicate that the social inequality in acceptance of publicly offered preventive services might decrease if municipalities adopt more proactive invitational procedures.

## Abbreviations

SES: Socio-Economic Status.

## Competing interests

The authors declare that they have no competing interests.

## Authors’ contributions

KA and MV conceived the study. YY developed the study design, performed the statistical analyses and drafted the manuscript. AE provided one variable that was used in the analyses. All authors contributed interpretation of results and helped to draft or critically revise the manuscript. All authors read and approved the final manuscript

## Pre-publication history

The pre-publication history for this paper can be accessed here:

http://www.biomedcentral.com/1471-2458/12/396/prepub
